# A Reliability Analysis of a MEMS Flow Sensor with an Accelerated Degradation Test

**DOI:** 10.3390/s23218733

**Published:** 2023-10-26

**Authors:** Qiaoqiao Kang, Yuzhe Lin, Jifang Tao

**Affiliations:** 1 Key Laboratory of Laser and Infrared System of Ministry of Education, Shandong University, Qingdao 266237, China; 202020457@mail.sdu.edu.cn; 2 School of Information Science and Engineering, Shandong University, Qingdao 266237, China; linyz@sdu.edu.cn; 3 Qingdao Xinnovis Microsystem Co., Ltd., Qingdao 266101, China

**Keywords:** flow sensor, accelerated degradation testing, reliability

## Abstract

With the wide application of flow sensors, their reliability under extreme conditions has become a concern in recent years. The reliability of a Micro Electro Mechanical Systems (MEMS) flow sensor under temperature ${(T}_{s})$ is researched in this paper. This flow sensor consists of two parts, a sensor chip and a signal-processing system (SPS). Firstly, the step-stress accelerated degradation test (SSADT) is implemented. The sensor chip and the flow sensor system are tested. The results show that the biggest drift is 3.15% for sensor chips under 150 °C testing conditions, while 32.91% is recorded for the flowmeters. So, the attenuation of the SPS is significant to the degeneration of this flowmeter. The minimum drift of the SPS accounts for 82.01% of this flowmeter. Secondly, using the Coffin–Manson model, the relationship between the cycle index and $T_{s}$ is established. The lifetime with a different $T_{s}$ is estimated using the Arrhenius model. In addition, Weibull distribution (WD) is applied to evaluate the lifetime distribution. Finally, the reliability function of the WD is demonstrated, and the survival rate within one year is 87.69% under 85 °C conditions. With the application of accelerated degradation testing (ADT), the acquired results are innovative and original. This research illustrates the reliability research, which provides a relational database for the application of this flow sensor.

## 1. Introduction

Flow sensors have been widely used in metrology and industry as a fundamental device [1,2,3]. They have received continuous attention and in-depth research with the development of Micro Electro Mechanical Systems (MEMS) technology [4,5,6]. With the progress of science and technology, efficient and intelligent flow sensors are often used in high-temperature or high-humidity environments [7,8,9,10]. Especially with the continuous development of MEMS flow sensors, the application field is more and more extensive. Unfortunately, their high performance and integrated functions receive superabundant attention, and their reliability tends to be forgotten. However, the MEMS sensor chip, as the core of the whole flow sensor system, suffers from degradation and a reduced reliability, which constitutes a major hazard to the safety performance of the system. Thus, the reliability of flow sensors under extreme conditions has been paid much more attention in recent years [11,12,13,14,15,16,17,18,19]. In addition, the reliability of electronic device systems, especially in regard to their performance with concerns for signal-processing system (SPS), has been reported very little, even though the SPS is one of the weakest components in the entire system [20,21,22]. Consequently, ensuring the reliability of flow sensors under extreme conditions cannot be ignored for their future operating characteristics and commercialization.

Accelerated degradation testing (ADT) has been widely applied as an efficient strategy for obtaining the reliability (life) information of assets in a shorter-than-normal period time by exposing the assets to higher-than-normal stresses [9]. The ADT is a good way of studying devices under extreme conditions, especially the step-stress accelerated degradation test (SSADT), which can set different temperatures during a finite test period time [10]. The performance degradation of high-temperature components, such as aero gas turbine engines and LEDs, has been illustrated, which was proved by experimental data [7,8,9,10,11,12,13,14,15]. However, the previous research has not considered the impacts caused by the stress conversion, or has assumed there is no effect. In recent years, the optimization of ADT has been considered, in which a drift parameter being a function of time and the lifetime distribution model have been studied. But the relationship of lifetime–stress and the accuracy of the life distribution model tend to get forgotten [15,16].

In this paper, the flow sensor consists of two parts, a MEMS sensor chip and a SPS. The SPS has lots of analog sub-parts such as amplifiers, comparators, filters, and digital–analog and analog–digital converters. The MEMS flow sensor’s lifetime and reliability are reported. Based on the SSADT of the temperature stress ($T_{s})$, the influences of $T_{s}$ on the MEMS sensor chip and SPS are discussed. The Coffin–Manson model is used to illustrate the relationship between the cycle index and $T_{s}$. The lifetime of the sensor chips is estimated with the Arrhenius model. Finally, Weibull distribution (WD) is applied to analyze the lifetime distribution. This paper is organized as follows. Section 2 elaborates on the experiment steps and methods. The experimental results of the SSADT are obtained and discussed in Section 3. Section 4 estimates the lifetime distribution and predicts the reliability with WD.

## 2. Materials and Methods

**Figure 1:**
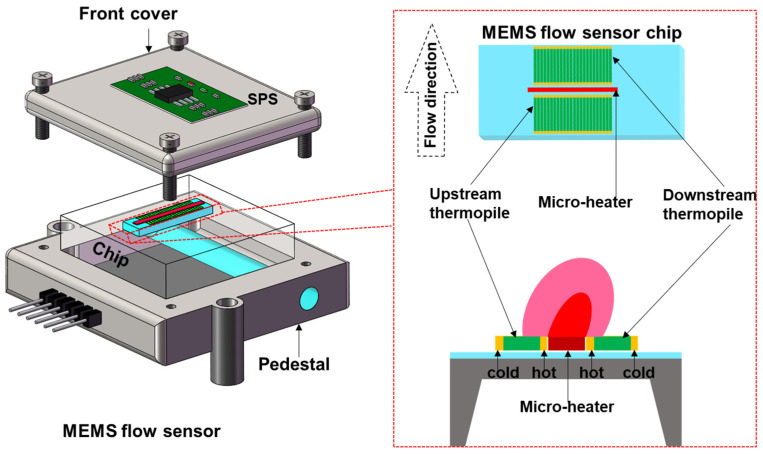
MEMS flow sensor.

As shown in Figure 1, the flow sensor consists of a MEMS flow sensor chip and the SPS (XINNOVIS GAS FLOW SENSOR MFA-40, Qingdao Xinnovis Microsystem Co., Ltd., Qingdao, China). The MEMS chip is fabricated between front cover and pedestal. The flow signal is transmitted to the SPS. The MEMS flow sensor includes three parts: a micro-heater, sensitive area, and silicon substrate. The heating structure (micro-heater) and sense structure (up/down-downstream thermopile) are made of polysilicon.

For this MEMS flow sensor, the physical principle of thermoelectric generation is based on Seebeck effect. As shown in Equation (1), the Seebeck coefficient is an important actor for this MEMS sensing chip.

(1) $$\alpha_{ab}=\frac{{d\Theta }_{ab}}{dT}(\mu V/K)$$

$\alpha_{ab}$ is a coefficient of Seebeck. $\Theta_{ab}$ is the potential (electric) force of Seebeck. T is the temperature of the micro-heater. When voltage is applied to the micro-heater, the micro-heater will generate heat to warm up the surroundings. The hot junctions of two thermopiles are heated. Then, initial thermoelectric voltage is generated. When there is air flow, the thermal field distribution is changed, leading to the initial voltage of the thermopile being changed accordingly [23,24,25,26,27].

**Figure 2:**
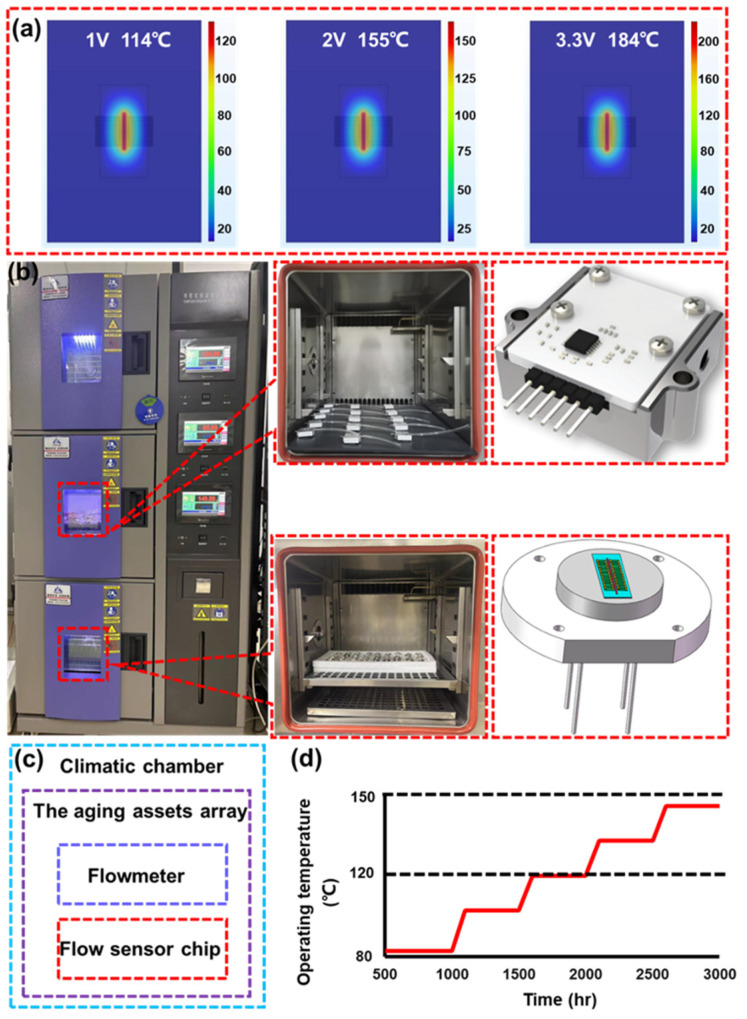
The steps of ADT. (**a**) Temperature distribution with different voltage; (**b**) Climatic and tested assets; (**c**) Flowchart of ADT. (**d**) Thermal cycle for ADT.

Before ADT, the resistance of the micro-heater (RHG) and the up-downstream thermopile (RUG, RDG) were tested separately. The average values (AVG) of the output voltage at 100 sccm and 500 sccm are shown in Table 1. The resistance of the resistors was measured, as shown in Table 2. The operation voltage was 3.3 V for the flow sensor. To obtain the operation temperatures with different input voltages and design the ADT, the finite element simulation was implemented. As shown in Figure 2a, the maximum temperature was 184 °C. But the SPS cannot operate under such a high temperature for a long time. Thus, to complete the accelerated experiment in a short time and obtain effective experimental phenomena and data, SSADT with 85–120–150 °C $T_{s}$ was performed.

As shown in Figure 2, the ADT was carried out on the flow sensor chip and flow sensor simultaneously, which was set up in a climatic chamber. Figure 2b–d show the assets in the climatic chamber. To obtain the output signal conveniently, the sensor chip was fabricated in a steel fixture, as shown in Figure 2a. The test period for every temperature gradient of SSADT was 500 h and each cycle took 3000 h, as shown in Figure 2d.

## 3. ADT Description

### 3.1. ADT Results

**Figure 3:**
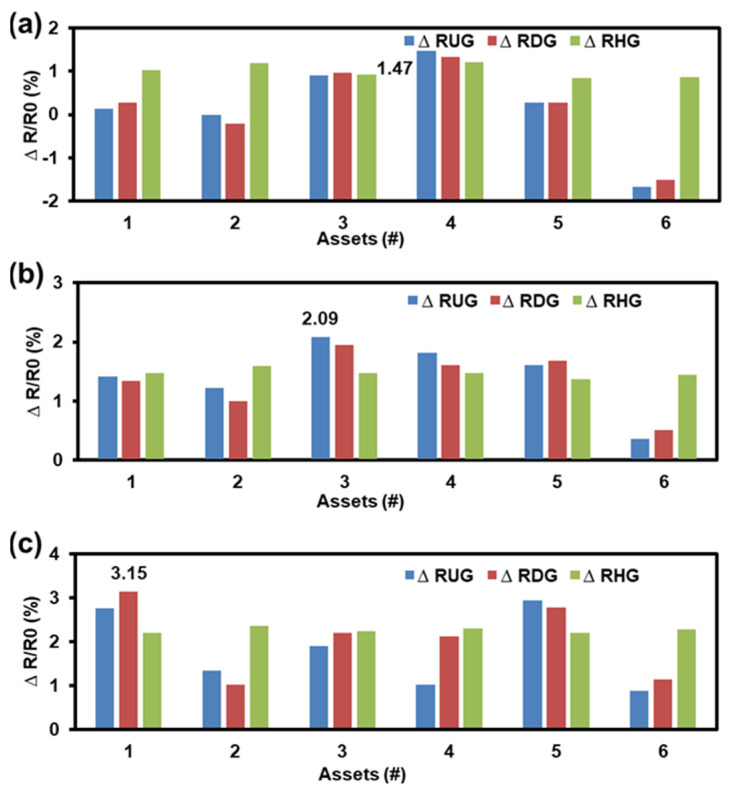
The ADT results of flow sensor chip with SSADT. (**a**) The maximum drift was 1.45% for 85 °C; (**b**) the maximum drift was 2.09% for 120 °C; and (**c**) the maximum drift was 3.15% for 150 °C.

As shown in Figure 1, considering the structure of this MEMS flow sensor, the output voltage changes were considered as the drifts of the characteristic parameters for the flow sensor, and the changes of the resistors were acquired as the drifts of the characteristic parameters for the sensor chip. The results of the drifts of the characteristic parameters were presented. Specifically, the resistance drifts of RHG, RDG, and RUG with 85 °C, 120 °C, and 150 °C $T_{s}$ were tested. This is shown in Figure 3a–c, where ΔR is the change in the resistor and $R_{0}$ is the initial resistor. After 3000 h of aging, the maximum drifts of 85 °C, 120 °C, and 150 °C were 1.35%, 2.09%, and 3.15%, respectively. It could be found that with the increases in $T_{s}$, the drift increased.

**Figure 4:**
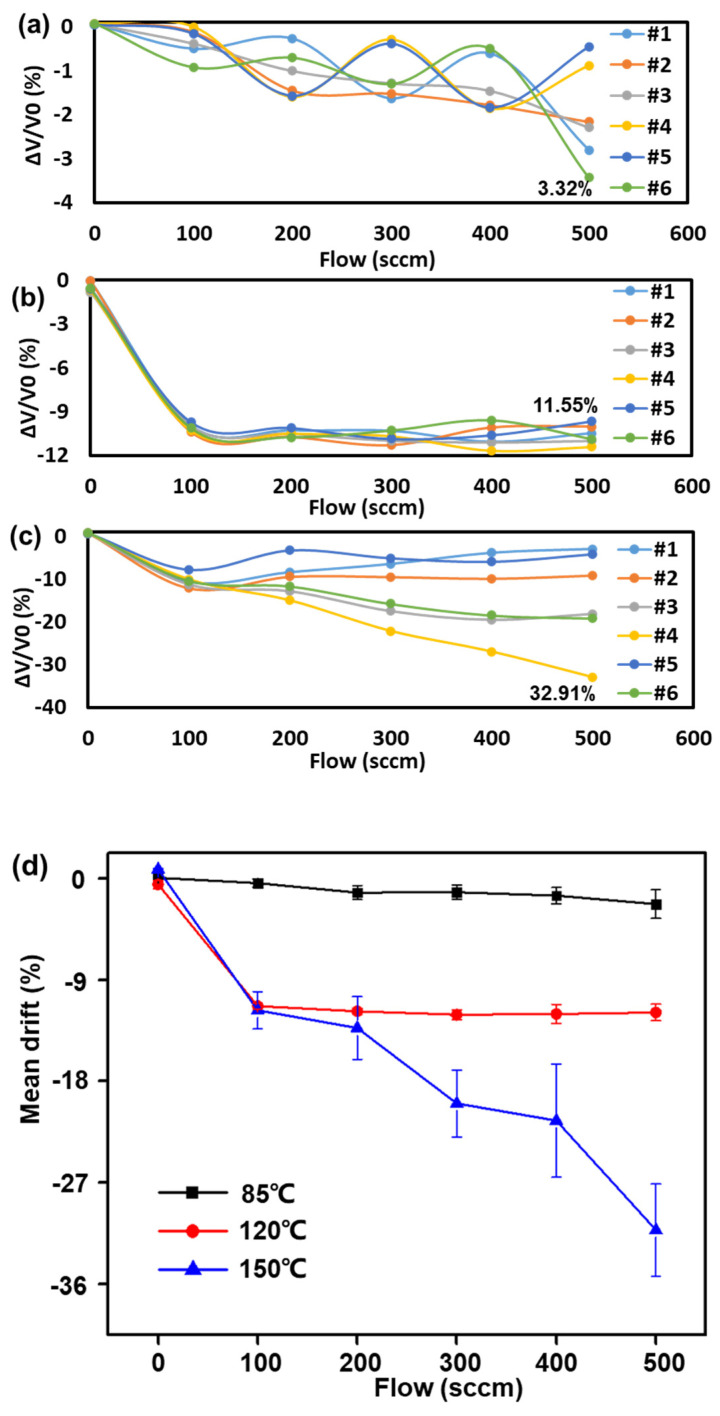
The ADT results of flow sensor with SSADT. (**a**) The maximum drift was 3.32% for 85 °C; (**b**) the maximum drift was 11.55% for 120 °C; and (**c**) the maximum drift was 32.19% for 150 °C. (**d**) The mean drift of 85 °C, 120 °C, and 150 °C.

For the flow sensors, the drifts were also tested. $V_{0}$ is the initial output voltage of various flow rates and ΔV corresponds to the changes. Comparing Figure 4a–d, from the #1–#6 samples, it can be found that the higher $T_{s}$, the stronger the attenuation. In addition, the attenuation value was turbulent with an increased $T_{s}$, and the larger flow rate was obviously enhanced. The biggest drift was −32.91% at 500 sccm with 150 °C.

To further analyze the attenuation of the SPS in the MEMS flow sensor system, the outputs of the SPS before and after the ADT were calculated. As shown in Table 3, taking the flow rate of 500 sccm as an example, ADT-C was the initial value of the MEMS flow chip and ADT-S was the initial value of the SPS. C-ADT was the aging value of the MEMS flow chip and S-ADT was the aging value of the SPS. In comparison, it was found that the attenuation of the SPS in the ADT was significantly greater than that of the MEMS flow chip. The minimum drift of the SPS accounted for 82.01% of the MEMS flow sensor system. Because the SPS was composed of comparators, analog operational amplifiers, and the digital signal-processing chip, there were fault overlap circumstances in the SPS fault diagnosis. Identifying the source of the attenuation is a complex task. But for this MEMS flow sensor system, the attenuation performance of the SPS was certified.

### 3.2. Modelling Description

Resistance tends to produce a thermal drift, which is a function of time t and temperature T [28,29,30]. Because there were thermal cycles in this SSADT, it was necessary to incorporate a Coffin–Manson component. The Coffin–Manson model reflects the fatigue failure of products under thermal sequential stress, and has also been successfully used to simulate the crack propagation process of solder joints subjected to temperature impact [31]. Thus, it can be used to describe the relationship between thermal fatigue failure and the temperature cyclic stress of products. The resistance drift based on Coffin–Manson dependency was proposed.

(2) $$\frac{∆R}{R_{0}}=exp\left[-\frac{∆E}{K_{B}}\cdot \left(\frac{1}{T_{1}}-\frac{1}{T_{2}}\right)\right]$$

(3) $$\varepsilon =\frac{A_{0}}{{∆T}^{\beta 1}}\cdot \frac{1}{f^{\beta 2}}\cdot exp\left[\frac{\Delta Ε}{K_{B}T_{max}}\right]$$

$K_{B}$ is the Boltzmann’s constant $8.36\times 10^{-5}eV{/}^{{}^{\circ}}K$. $T_{max}$ is the maximum temperature. ΔΕ is the activation energy of two different ADT stresses. ∆R is the drift. t is the time of the ADT. $T_{1}$ and $T_{2}$ are different environmental temperatures. ε is the cycle index. ∆T is the operation temperature. f is the cycle frequency. $A_{0}$, β1, and β2 are undetermined constants.

Based on Figure 3 and Figure 4, the drift rate can be calculated. For the MEMS sensor chip, we can obtain the ΔΕ of assets with different stresses via the drift. Furthermore, the drift of RHG was more stable with a maximum average drift rate in various asset groups. Consequently, the activation energy of each group of tested assets can be acquired by calculating the RHG drift rate with different ADT conditions. Then, the activation energies of 85 °C (${∆E}_{25{}^{\circ}C-85{}^{\circ}C}$), 120 °C (${∆E}_{25{}^{\circ}C-120{}^{\circ}C}$), and 150 °C (${∆E}_{25{}^{\circ}C-150{}^{\circ}C}$) of the sensor chip were obtained using Equation (4), with maximum average drifts of 1.01%, 1.47%, and 2.04%, respectively.

(4) $$\begin{array}{c} {∆E}_{\left(T_{1}-T_{2}\right)}=\frac{8.36\;\times \;10^{-5}\;eV/K\cdot ln\left(\frac{∆R_{RHG}}{R_{RHG0}}\right)}{\frac{1}{T_{1}}-\frac{1}{T_{2}}}\\ {∆E}_{25{}^{\circ}C-85{}^{\circ}C}=\frac{8.36\;\times \;10^{-5}\;eV/K\cdot ln\left(1.01\right)}{\frac{1}{298\;K}-\frac{1}{358\;K}}=0.0015\;eV\\ {∆E}_{25{}^{\circ}C-120{}^{\circ}C}=\frac{8.36\;\times \;10^{-5}\;eV/K\cdot ln\left(1.47\right)}{\frac{1}{298\;K}-\frac{1}{393\;K}}=0.039\;eV\\ {∆E}_{25{}^{\circ}C-150{}^{\circ}C}=\frac{8.36\;\times \;10^{-5}\;eV/K\cdot ln\left(2.04\right)}{\frac{1}{298\;K}-\frac{1}{423\;K}}=0.060\;eV \end{array}$$

Meanwhile, the parameter of Coffin–Manson was obtained with the ΔΕ of the SSADT. As shown in Table 4, the value was 2.303 for $A_{0}$, 0.154 for β1, and 0.187 for β2. Based on the above research, the ∆E of different SSADTs of this flow sensor can be estimated without having to calculate the ∆R.

Similarly, the $E_{a}$ of this MEMS flow sensor with different stresses via the drift can be obtained. Thus, the activation energy can be calculated with the maximum average drifts of 1.21%, 11.15%, and 16.82%, respectively, with a flow rate of 500 sccm. Then, the activation energies of 85 °C (${∆E}_{25{}^{\circ}C-85{}^{\circ}C}$), 120 °C (${∆E}_{25{}^{\circ}C-120{}^{\circ}C}$), and 150 °C (${∆E}_{25{}^{\circ}C-150{}^{\circ}C}$) of the flow sensor were obtained using Equation (5), with maximum activation energies of 0.028 eV, 0.359 eV, and 0.421 eV, respectively. In the same way, the parameter of the Coffin–Manson of the flow sensor was obtained with the ΔΕ of the SSADT. As shown in Table 5, the value was 1.764 for $A_{0}$, 0.235 for β1, and 0.298 for β2, as shown in Table 5.

$$\begin{array}{c} {∆E}_{25{}^{\circ}C-85{}^{\circ}C}=\frac{8.36\;\times \;10^{-5}\;eV/K\cdot ln\left(1.21\right)}{\frac{1}{298\;K}-\frac{1}{358\;K}}=0.028\;eV\\ {∆E}_{25{}^{\circ}C-120{}^{\circ}C}=\frac{8.36\;\times \;10^{-5}\;eV/K\cdot ln\left(11.15\right)}{\frac{1}{298\;K}-\frac{1}{393\;K}}=0.249\;eV\\ {∆E}_{25{}^{\circ}C-150{}^{\circ}C}=\frac{8.36\;\times \;10^{-5}\;eV/K\cdot ln\left(16.82\right)}{\frac{1}{298\;K}-\frac{1}{423\;K}}=0.238\;eV \end{array}$$

According to the ∆E obtained by the drifts, the lifetime can be estimated. ε was obtained from the Coffin–Manson modeling. Specifically, the Arrhenius lifetime model was employed in the degradation model, which has been widely used in the reliability literature [31,32,33,34].

(5) $$N_{1}=\frac{l_{1}}{{l_{1}}^{\prime }}=exp\left[\frac{∆E}{K_{B}}\cdot \left(\frac{1}{S1}-\frac{1}{S^{\prime }}\right)\right]$$

**Figure 5:**
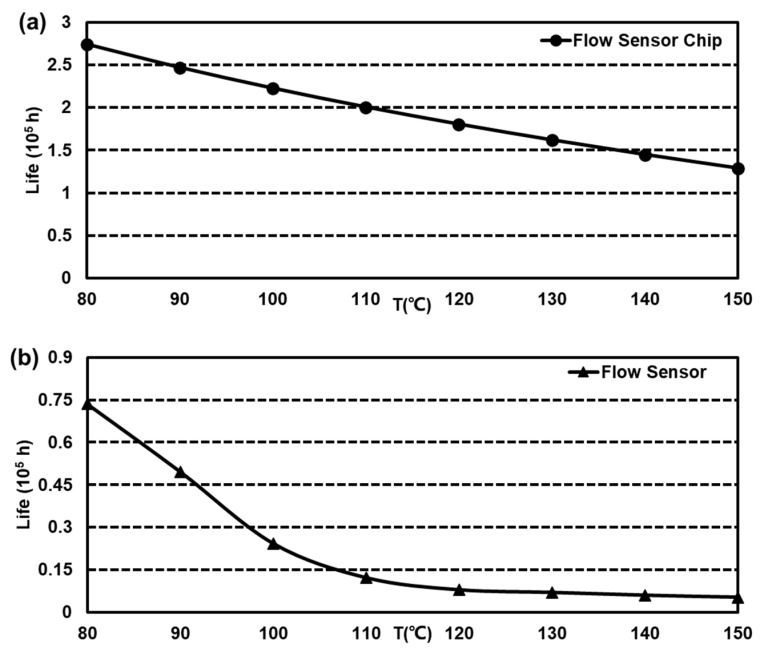
The ADT results of lifetime–stress. (**a**) The lifetime–stress of MEMS sensor chip and (**b**) the lifetime–stress of MEMS sensor.

$N_{1}$ is the Arrhenius accelerator. $l_{1}$ is the lifetime of S1. ${l_{1}}^{\prime }$ is the lifetime of $S^{\prime }$. The lifetimes under various conditions can be estimated. For this MEMS flow sensor system, the lifetime was calculated, as shown in Table 6. It can be found that the lifetime shrunk more obviously from 8.290 years to 0.594 years in the range from 85 °C to 150 °C. But for the sensor chip, the change was from 29.702 to 14.724 years. In addition, the lifetime of other conditions was acquired with obtained lifetime–stress data fitting. As is shown in Figure 5, the predicted lifetime distributions are presented.

## 4. Reliability Discussion

Reliability engineering has become a powerful tool for determining the behavior of devices over time. One of the most ingrained concepts within reliability is the description of survival rate through lifetime distribution modeling. For further exploration, it was necessary for us to evaluate the lifetime distribution. As we can see from the introduced publications, there are many methods for the description of lifetime distribution: exponential distribution (ED), Weibull distribution (WD), and Gamma and Gaussian distribution [35,36,37,38]. Each lifetime distribution model contains a cumulative distribution function and a reliability function.

Considering numerous versions and types, we selected the WD model to analyze the reliability of the flow sensors. WD modifications were proposed through diverse mathematical techniques to ensure that the behavior described was closest to most devices [36,37,38]. The cumulative distribution function F(t) and the reliability function R(t) can be calculated. The reliability-related parameters of the F(t) and R(t) are crucial adjectives.

(6) $$F\left(t\right)=1-\exp\left[-{\left(\frac{t}{\alpha_{0}}\right)}^{\beta }\right]$$

(7) $$R\left(t\right)=\exp\left[-{\left(\frac{t}{\alpha_{0}}\right)}^{\beta }\right]$$

**Figure 6:**
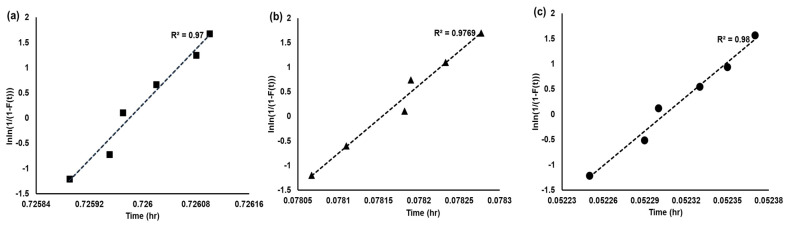
The fitting results of MEMS flow sensor with WD distribution. (**a**) The WD distribution result of 85 °C; (**b**) The WD distribution result of 120 °C; and (**c**) The WD distribution result of 180 °C.

$\alpha_{0}$ is the scale factor or characteristic function. β is the shape factor or shape parameter. $\alpha_{0}$ and β and can be obtained by a fitting procedure [37,38]. t is the working time. After obtaining the modeling, the results were analyzed, as shown in Figure 6a–c. The log time of the acceleration was displayed in the horizontal coordinate axis and the ln[−ln(1 − F(t)] in the vertical axis. The results at each stress level were fit in straight lines. Therefore, it can be considered that the failure time of this flow sensor obeyed WD under different temperature stresses. The R-squared of 85 °C, 120 °C, and 150 °C are displayed in Table 7. In addition, $\alpha_{0}$, β, and the $R\left(t\right)$ were acquired, respectively, with a 3000 h aging time. As shown in Table 7, the $R\left(t\right)$ of this flow sensor was 87.69% with 85 °C. At the same time, the $R\left(t\right)$ was 32.77% for 120 °C and 19.39% for 150 °C. Thus, the reliability of this flow sensor with different $T_{s}$ was acquired.

## 5. Conclusions

In this paper, the reliability of a MEMS flow sensor with $T_{s}$ was estimated. The degradation characteristics of the MEMS flow sensor chip and the flow sensor with SSADT were acquired. The biggest drifts of 85 °C, 120 °C, and 150 °C were 1.35%, 2.09%, and 3.15% for the MEMS flow sensor chip, respectively. For the flow sensor, the biggest drift was −32.91% at 500 sccm with 150 °C. It could be found that the attenuation of the SPS was significant. The minimum drift of the signal-processing system accounted for 82.01% of the whole system. To obtain the lifetime of this flow sensor, the activation energies of 85 °C,120 °C, and 150 °C were calculated. The maximum activation energies were 0.028 eV, 0.359 eV, and 0.421 eV, respectively. By using the Coffin–Manson model, the relationship between the cycle index and $T_{s}$ with SSADT is established. The lifetime of the MEMS flow sensor chip and flow sensor with different $T_{s}$ was estimated using the Arrhenius model. Furthermore, the reliability with WD was demonstrated. The $R\left(t\right)$ was 87.69% for 85 °C, 32.77% for 120 °C, and 19.39% for 150 °C.

To sum up, the content illustrates the reliability study of a MEMS flow sensor with $T_{s}$. The results are innovative and original, and lay a foundation for this MEMS flow sensor to work under an extreme temperature environment. Based on this research, the reliability database is set up. Moreover, we will further analyze the causes of degradation to instruct the design of MEMS flow sensor systems and make them more practical in circumstances with complex conditions.

## Figures and Tables

**Table 1:** Repeatability and consistency of flow sensor.

Flow	Index	#1	#2	#3	#4	#5	#6
100 sccm	Avg (V)	1.868	1.873	1.856	1.863	1.844	1.867
500 sccm	Avg (V)	4.801	4.856	4.885	4.894	4.872	4.893

**Table 2:** Repeatability and consistency of flow sensor chip.

	Index	#1	#2	#3	#4	#5	#6
RUG	Avg (KΩ)	119.7	118.1	122.5	121.1	121.3	115.8
RDG	Avg (KΩ)	116.5	122.2	114.7	120.2	122.8	120.2
RHG	Avg (Ω)	704.3	703.6	704.5	705.3	704.6	703.7

**Table 3:** The ADT results of flow sensor and SPS.

Index	#1	#2	#3	#4	#5	#6
ADT-C (mV)	9.533	9.752	9.634	9.607	9.611	9.664
C-ADT (mV)	9.529	9.751	9.621	9.603	9.591	9.657
Drift (%)	17.29	0.81	1.37	1.925	17.98	3.43
ADT-S (V)	4.801	4.856	4.894	4.893	4.893	4.816
S-ADT (V)	4.778	4.733	3.943	4.685	4.782	4.612
Drift (%)	82.70	99.19	98.63	98.07	82.01	96.56

**Table 4:** The parameter estimation of Coffin–Manson results of flow sensor chip.

Index	ε	f	∆T(K)	$T_{max}(K)$	ΔΕ(eV)	$A_{0}$	β1	β2
25 °C–85 °C	2	0.5	60	85	0.0015	2.303	0.154	0.187
25 °C–120 °C	2	0.5	95	120	0.039	2.303	0.154	0.187
25 °C–150 °C	2	0.5	125	150	0.060	2.303	0.154	0.187

**Table 5:** The parameter estimation of Coffin–Manson results of flow sensor.

Index	ε	f	∆T(K)	$T_{max}(K)$	ΔΕ (eV)	$A_{0}$	β1	β2
25 °C–85 °C	2	0.5	60	85	0.028	1.764	0.235	0.298
25 °C–120 °C	2	0.5	95	120	0.249	1.764	0.235	0.298
25 °C–150 °C	2	0.5	125	150	0.238	1.764	0.235	0.298

**Table 6:** The estimation of lifetime.

Index	Tem (°C)	$E_{a}$ (eV)	Lifetime (Years)
Sensor chip—RHG	85 °C	0.0015 eV	29.702
	120 °C	0.039 eV	20.548
	150 °C	0.060 eV	14.724
Flow sensor—500 sccm	85 °C	0.028 eV	8.290
	120 °C	0.249 eV	0.893
	150 °C	0.238 eV	0.594

**Table 7:** The parameter estimation of reliability.

Index	85 °C	120 °C	150 °C
$\alpha_{0}$ (${\times 10}^{5}$)	0.726	0.0781	0.0523
β	0.96	0.96	0.96
$R\left(t\right)$ (%)	87.69	32.77	19.39

## Data Availability

All data supporting reported results are included in the manuscript.
